# Plant-Derived Bioactive Compounds in Colorectal Cancer: Insights from Combined Regimens with Conventional Chemotherapy to Overcome Drug-Resistance

**DOI:** 10.3390/biomedicines10081948

**Published:** 2022-08-11

**Authors:** Laura Ioana Gavrilas, Daniel Cruceriu, Andrei Mocan, Felicia Loghin, Doina Miere, Ovidiu Balacescu

**Affiliations:** 1 Department of Bromatology, Hygiene, Nutrition, “Iuliu Hatieganu” University of Medicine and Pharmacy, 23 Marinescu Street, 400337 Cluj-Napoca, Romania; 2 Department of Functional Genomics, Proteomics and Experimental Pathology, “Prof. Dr. Ion Chiricuta” Oncology Institute, 34-36 Republicii Street, 400015 Cluj-Napoca, Romania; 3 Department of Molecular Biology and Biotechnology, “Babes-Bolyai” University, 5-7 Clinicilor Street, 400006 Cluj-Napoca, Romania; 4 Department of Pharmaceutical Botany, “Iuliu Hatieganu” University of Medicine and Pharmacy, 23 Marinescu Street, 400337 Cluj-Napoca, Romania; 5 Department of Toxicology, “Iuliu Hatieganu” University of Medicine and Pharmacy, 400337 Cluj-Napoca, Romania

**Keywords:** combination therapy, chemotherapy resistance, colorectal cancer, drug resistance, curcumin, resveratrol, epigallocatechin gallate

## Abstract

Acquired drug resistance represents a major clinical problem and one of the biggest limitations of chemotherapeutic regimens in colorectal cancer. Combination regimens using standard chemotherapeutic agents, together with bioactive natural compounds derived from diet or plants, may be one of the most valuable strategies to overcome drug resistance and re-sensitize chemoresistant cells. In this review, we highlight the effect of combined regimens based on conventional chemotherapeutics in conjunction with well-tolerated plant-derived bioactive compounds, mainly curcumin, resveratrol, and EGCG, with emphasis on the molecular mechanisms associated with the acquired drug resistance.

## 1. Introduction

Colorectal cancer (CRC) prevalence remains remarkably high in both men and women, being one of the leading causes of cancer deaths despite all efforts made in recent years in screening and treatment development [1]. Although surgery is the main treatment option for patients with likely curable CRC, depending on the disease stage, neoadjuvant chemotherapy and/or radiotherapy may be used before or after surgery [2]. Despite these efforts, up to 30% of the patients with stage I–III and about 65% of patients with stage IV CRC will develop recurrent disease [3]. In most cases, these conventional treatments are associated with high tumor relapse and recurrence rates, especially due to the acquired drug resistance [4]. Therefore, the CRC-acquired drug resistance represents a major clinical issue and the most significant limitation of chemotherapeutic regimens available nowadays, placing more emphasis on the need for novel, safe, and more effective approaches.

The therapeutic potential of bioactive natural compounds in different types of cancers, including breast cancer [5], prostate cancer [6], glioblastoma [7], and CRC [8], among others, especially in combination with other conventional chemotherapeutic agents, has been widely investigated, with mainly positive outcomes. Combination regimens using standard chemotherapeutic agents and bioactive natural compounds derived from food or plants may be one of the most valuable strategies to overcome drug resistance. Combination regimens refer to the simultaneous administration of a conventional drug with one natural agent or a mixture of those, as well as the co-treatment of two or more natural compounds [9,10,11]. The available research indicates that plant-derived bioactive products can increase drug efficacy at a lower dose, thus, reducing dosage toxicity [10,11]. In addition, they can re-sensitize chemoresistant cells by multiple molecular mechanisms representing an emerging area of cancer research. The plant-derived compounds most studied in relation to their capacity to potentiate other conventional chemotherapeutic drugs in CRC, especially by blocking and/or reversing the acquired drug resistance mechanisms, are curcumin (diferuloylmethane), resveratrol (3,4’,5-trihydroxystilbene), and (-)-Epigallocatechin gallate (EGCG). Due to the extensive data supporting curcumin, resveratrol, and EGCG efficacy in re-sensitizing chemoresistant cells, as well as the promise revealed by the latest research on new formulations with increased bioavailability, these natural compounds are regarded as leading candidates to counteract acquired drug-resistance in the near future in clinical practice. 

Curcumin represents the most important polyphenolic active ingredient derived from turmeric (the rhizomes of *Curcuma longa*). Extensive research has highlighted the potential of curcumin in the prevention and therapy of colorectal cancer due to its anti-inflammatory, antioxidant, and anticarcinogenic properties. Recent studies have emphasized that combining conventional chemotherapeutic agents and curcumin may effectively overcome drug resistance [11,12]. Curcumin has been confirmed as safe and non-toxic in humans, when administered at doses up to 10 g/day [13]. However, curcumin undergoes extensive metabolism in the liver and demonstrates poor bioavailability, thus accounting as a main problem towards its chemosensitizing potential [14]. Approaches to enhance curcumin bioavailability includes nano-encapsulation [15] or association with piperine, which act as a bio-enhancers that considerably improve the absorption of curcumin by up to 2000-fold [16].

Resveratrol is a phytoalexin found in grapes skin, wine, berries, and other plant sources, mainly produced in response to bacterial or fungi infections as a defense mechanism [17,18]. It is a well-studied bioactive dietary compound known to modulate several cancer-related molecular pathways, with promising applications as an adjuvant in cancer management. Accumulating evidence has pointed out the efficacy of resveratrol as a chemosensitizer in multiple cancer types, including CRC [10,12]. Resveratrol is easily absorbed and transformed, primarily into sulfo- and glucuro-conjugates that are excreted in the urine. Resveratrol appears to be well tolerated, and no overt toxicity in humans has been reported [19]. However, these findings are still sporadic, and many areas demand more research and clarity. Specifically, the precise make-up of the endogenous metabolites, their biological properties, as well as the nature of interaction with other drugs or bioactive dietary components.

EGCG represents the most valuable green tea polyphenol with growth inhibitory properties against many tumors, including CRC [20]. It has been proposed that EGCG can enhance drug sensitivity in various cancers, such as gastric cancer [21], hepatocellular carcinoma [22], and CRC [23,24]. In addition, it is widely recognized as an important chemopreventive agent, as well as a modulator of apoptosis, angiogenesis, or other cancer-related biological processes [20]. Research advances in bioavailability studies involving absorption and metabolic biotransformation of tea catechins reported that the observed discrepancy between in vitro and in vivo investigations is mostly due to the low bioavailability of green tea catechins. Stability and absorption rate, as well as efflux, influence their bioavailability. However, molecular modification, drug delivery systems based on nanostructures, and co-administration with some other bioactive ingredients can all improve the bioavailability of catechins [25]. Despite the health beneficial effects reported in numerous studies, food supplements containing green tea catechins should be administered with caution as exposure to green tea extracts at and above 800 mg EGCG/day in intervention studies causes elevated serum transaminases, which is indicative of liver injury [26].

Therefore, in the present paper, we analyze how the combination of conventional chemotherapeutics with bioactive dietary compounds, mainly curcumin, resveratrol, and EGCG, may contribute to a better response of CRC by reversing epithelial to mesenchymal transition (EMT), re-sensitizing chemoresistant cells, and enhancing the apoptosis. In this regard, we conducted a piece of literature research on Pubmed, ScienceDirect, and Google Scholar and selected publications between January 2000 and April 2022, using the search terms “colorectal cancer”, “drug resistance”, “chemotherapy resistance”, “combination therapy”, “combinatorial treatment”, “synergistic effect” “bioactive dietary compounds”, ”natural compounds” “chemosensitization”, “curcumin”, “resveratrol”, “epigallocatechin-3-gallate (EGCG)” individually or in combination. We included original research and review articles on both animals and humans. The non-English publications were excluded.

## 2. Colorectal Cancer: Epidemiology, Risk Factors, Therapeutic Approach

An active lifestyle, together with healthy eating patterns and particular natural components from food, can prevent, delay, and even reverse chronic diseases, especially those associated with cancer [27,28,29,30]. Functional foods, such as fish-rich in ώ-3-polyunsaturated fatty acids, algae, fiber-rich foods or medicinal mushrooms, as well as bioactive dietary compounds, such as curcumin, resveratrol, epigallocatechin-3-gallate (EGCG), quercetin, α-mangostin, and vitamin D, have been reported to modulate important pathways involved in cancer prevention and treatment [7,8,10,11].

Current epidemiologic data indicate that CRC continues to be the third most diagnosed type of cancer but second in terms of mortality, with an estimated incidence of 1.9 million new cases and 935,000 deaths in 2020 [1]. CRC is recognized as a hallmark of socioeconomic improvement, although in most developed regions, rates have decreased over the past decades due to improved screening programs and advanced treatment approaches. Nonetheless, its incidence showed a steadily rising pattern in developing countries in conjunction with changes in environmental factors, including poor dietary choices, sedentary lifestyle, obesity, smoking, and alcohol consumption, all confirmed as critical determinants of CRC risk.

Since it’s a lifestyle-related cancer, dietary patterns play an essential role in developing CRC. As support in this regard, the International Agency for Research on Cancer classified a high intake of processed meat as carcinogenic to humans [31]. Additional dietary risk factors include common patterns of a Western diet, such as high intakes of red meat, refined carbohydrates (white pasta, refined rice, or white flour), sweets and sugar-sweetened beverages. In contrast, consumption of vegetables, legumes, and fruits rich in bioactive dietary components, as well as options of fiber-rich foods and fish are negatively associated with CRC risk and sustain a normal weight status and a healthy gut microbiota [32,33,34]. In addition to dietary intake, regular exercise is one of the most important lifestyle factors for CRC prevention [35].

Conventional strategies for colorectal cancer treatment include surgery, radiotherapy, immunotherapy, and chemotherapy. The most common therapeutic approach is surgery with chemotherapy alone or in combination with radiotherapy, given before or after surgery to most patients, depending on the disease stage [2]. Therapeutic regimens include one or more chemotherapeutic agents, such as platinum derivates (oxaliplatin, cisplatin), antimetabolites (5-fluorouracil, capecitabine), topoisomerase inhibitors (irinotecan), and more recently the targeted therapies with bevacizumab, cetuximab, and panitumumab, chosen based on the molecular profile of the patient [2,36,37]. The first line approach in advanced CRC includes 5-fluorouracil (5-FU) with folinic acid alone or combined with oxaliplatin (FOLFOX), while irinotecan represents a first-line therapeutic approach for metastatic colorectal cancer alone or combined with 5-FU [38]. The main cause of treatment failure and the need of switching between several chemotherapeutic regimens is mainly attributed to the development of drug resistance in tumor cells.

## 3. Mechanisms of Acquired Drug Resistance in CRC

Chemotherapeutic regimens used for CRC treatment lead to severe adverse effects that alter the quality of life of cancer patients, impair the treatment’s course and the treatment outcome, leading to malnourishment and depression [39]. However, the biggest limitation of systemic chemotherapeutic regimens available at the moment is the inability to prevent acquired drug resistance, with many patients experiencing relapsed/recurrent disease refractory to a variety of drugs, thus leaving metastatic patients with no treatment options [4]. Two types of drug resistance, baseline and acquired, are responsible for chemoresistance and treatment failure. [40,41]. The molecular mechanisms associated with acquired drug resistance, occurring during treatment, are related to increased rates of drug efflux, evasion of apoptosis, induction of the epithelial-mesenchymal transition (EMT), and persistence of cancer stem cells [41,42]. The activation of these mechanisms is mainly mediated by mutations, alterations, alternative splicing, and post-translational modifications of the genes and proteins that belong to them.

Several proteins involved in drug efflux belonging to ABC family transporters: ABCB1 (P-glycoprotein-Pgp), ABCC1 (MDR-associated protein 1-MRP1), and ABCG2 (breast cancer resistance protein-BCRP) are increased in colon cancer. These transporter proteins, by efflux the drugs outside the cancer cells, decrease the intracellular concentration of the drug and, therefore, their therapeutic effects [41,43]. Consequently, one practical approach to increase the drug efficacy is to maintain the intracellular concentration of the drug by inhibiting these proteins and re-sensitize resistant cells by using bioactive natural compounds.

EMT, another biological mechanism associated with chemoresistance, implies the transformation of epithelial cells to mesenchymal cells in specific pathological circumstances and represents the first step in tumor invasion and metastasis [4]. The process of EMT involves molecular reprogramming of the cells, including loss of cell adhesion molecules, such as E-cadherin, which changes the epithelial cell morphology, as well as overexpression of N-cadherin, osteopontin, Snail1, Snail2 (slug) and other interstitial proteins, thus, facilitating tumor cells to obtain higher potential with respect to migration, invasion, anti-apoptotic potential, and the degradation of extracellular matrix [4,44]. Therefore, EMT is a key event during the early stages of invasion and metastasis and on the onset of drug resistance.

Chemoresistance is also characterized by the ability of cancer cells to survive by suppressing cell death. A high grade of the alteration of cancer cell death-related pathways, resulting in decreased cell death rates, appear during the malignant transformation of colonic tissues into CRC [4]. In cases of apoptosis, the most documented alterations concern the Bcl-2 family and caspase genes [4]. Finally, yet importantly, the formation of cancer stem cells (CSCs) is a well-documented hallmark of chemoresistance [45]. CSCs, which represent a small subpopulation of self-renewal epithelial cancer cells, can be induced from differentiated cancer cells as a response from therapeutic pressures. CSCs are generally characterized by the expression of surface markers associated with stem cells, such as CD133, CD44, CD90 [45,46].

Although, tumors develop resistance to chemotherapeutic drugs either by decreasing the intratumor level of the drug or by genetic and epigenetic modifications within the cancer cells, which can alter apoptosis mechanisms and favor EMT, the resistance mechanisms are, of course, unique to each treatment. Specifically, nuclear factor-erythroid 2-related factor 2 (Nrf2), a key regulator of cellular defense against oxidative and electrophilic stresses, is considered significant in 5-FU resistance [47]. In addition, irinotecan administration modulates the expression and/or the activation of multiple proteins involved in the EGFR-MAPK7 [48], NFkB8 [49], and PI3K-AKT/mTOR9 [50] signaling pathways. These irinotecan-triggered gene expression alterations are actually promoting cell survival, proliferation, and invasiveness, thus being important contributors in the development of irinotecan resistance.

## 4. Combined Approaches in CRC Treatment Using Bioactive Natural Compounds

Combination regimens account for the simultaneous targeting of many cancer pathways having pleiotropic effects, allowing the reduction in the development of tumor drug resistance, as well as re-sensitizing chemoresistant cells. In most cases, plant-derived bioactive components additionally target multiple apoptotic mechanisms being much more effective than the use of single drug treatment.

**Figure 1:**
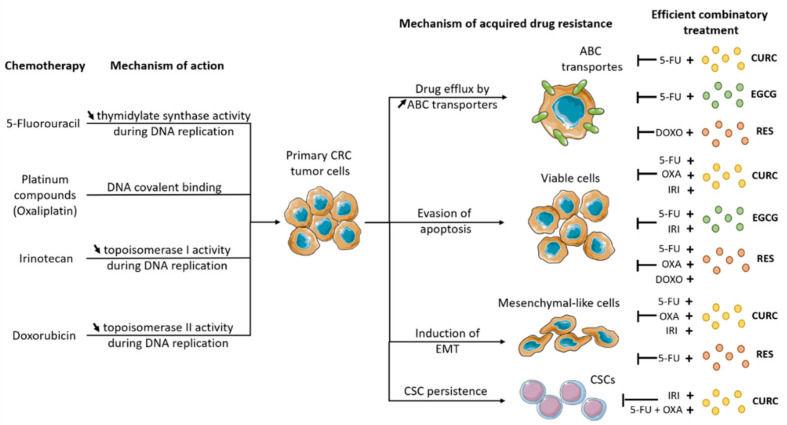
Combinatory therapeutic strategies involving a systemic chemotherapeutic drug and a plant-derived compound that proved their efficiency in reversing the acquired drug resistance in CRC. OXA—oxaliplatin; 5-FU—5-fluorouracil; IRI—irinotecan: DOXO—doxorubicin; CURC—curcumin; EGCG—epigallocatechin gallate; RES—resveratrol; ↓—increased, ↑—decreased.

The main mechanisms by which CRC tumor cells acquire drug resistance in return to systemic chemotherapy, along with the most important combinatory therapies involving a plant-derived compound that might re-sensitize the cancer cells are presented in Figure 1.

## 5. 5-FU

The most extensively used chemotherapeutic agent in colorectal cancer regimens is represented by 5-Fluorouracil (5-FU). The fluoropyrimidine analogue, 5-FU, is an antimetabolite drug with anticancer activity inhibiting the thymidylate synthase (TS) during DNA replication. Despite multiple advantages, the clinical use of 5-FU is limited due to the development of drug resistance [51].

He and colleagues confirmed that curcumin can enhance the sensitivity of HCT-8 colorectal cancer cells to 5-FU by down-regulating HSP-27 and P-gp protein, a well-known MDR-protein from the ABC family, both correlated with MDR [52]. In addition, another study demonstrated that curcumin can boost the anticancer activity of the 5-FU due to the suppression of *MDR1*. ROS induction mediated this action mechanism through modulation of the miR-27a-ZBTB10-Sp-axis [53]. Moreover, curcumin could inhibit proliferation and enhance apoptosis in 5-FU-resistant colorectal cell lines modulating a group of EMT-suppressive miRNAs [54]. The same authors further demonstrated that treatment with curcumin sensitized 5-FU to promote tumor growth inhibition in vivo in a xenograft mouse model [54]. Curcumin has been included in phase I and phase II clinical trials with FOLFOX showing anti-proliferative effects, as well as proving safe and tolerable adjunct to FOLFOX chemotherapy in patients with colorectal cancer at doses up to 2 g daily [55,56].

In DLD-1 and SW480 colorectal cancer cell lines, EGCG associated with 5-FU resulted in synergistic growth suppression, as showed by the combination index value of less than 1.0 [24]. More recently, Xiaoquin et al. demonstrated that EGCG enhanced the chemo-sensitivity of 5-FU at low doses in HCT-116 and DLD1 CRC cells by inhibiting cancer proliferation, targeting apoptosis and DNA damage [23]. The underlying mechanism is associated with the overexpression of miR-155-5p, which in turn strongly suppresses target gene *MDR1* expression, blocking the efflux of 5-FU, ultimately leading to cancer cell apoptosis.

A recent research conducted by Chung and colleagues [57] demonstrated the pleiotropic potential of the combined treatment with resveratrol and 5-FU in CRC. The combination regimen enhanced the antiproliferative potential of 5-FU in colorectal cancer by targeting cell cycle arrest in S-phase and augmented 5-FU pro-apoptotic effect by simultaneously inhibiting Akt and STAT3 signaling pathways. Furthermore, the combination of the two agents repressed EMT transition, targeting expression levels of slug and vimentin, both being downregulated following the combinatorial treatment. Remarkably, the combined approach with resveratrol and 5-FU-inhibited STAT3, a well-known transcription factor, from binding with the promoter region of *hTERT* gene, demonstrating the anti-telomerase activity of the regimen. Likewise, in HT-29 and SW-620 CRC cell lines, the treatment regimen based on 5-FU, combined with resveratrol, resulted in enhanced reactive oxygen species (ROS) and lipid peroxides, which was linked to the inhibition of oncogenic proteins AKT and STAT3 [58]. Moreover, it has been demonstrated in a recent study that resveratrol can chemosensitize 5-FU-resistant CRC cells in a tumor microenvironment suppressing the pro-carcinogenic and metastatic potential of tumor necrosis factor-β (TNF-β). It was noteworthy that resveratrol treatment strongly modulated EMT factors, showing significant suppression of vimentin and slug while increasing E-cadherin [59].

As summarized in Table 1, numerous studies have highlighted the potential of curcumin and other naturally derived compounds to enhance the drug response rates and overcome the drug resistance of most of the chemotherapeutic agents used in CRC treatment, including curcumin.

## 6. Platinum Compounds

The platinum-based compounds, such as cisplatin, oxaliplatin, and carboplatin, have been widely used in oncology for the last 40 years in treating several cancers, including bladder, ovarian, testicular, lung, head and neck, and colorectal cancer. The mechanism of action of most platinum-based drugs involves covalent binding to purine DNA bases leading to the formation of DNA adducts, thereby inhibiting DNA replication and transcription finally leading to apoptotic cell death [77,78]. The major limitations for the use of these chemotherapeutic agents are the frequent onset of drug resistance and the common side effects associated with toxicity [78]. The use of naturally derived compounds together with platinum-derived agents presents synergic effects increasing the efficiency of the conventional treatment, which may lead to dose reduction, as well as the decline in the adverse effects of chemotherapy.

### 6.1. Cisplatin

Cisplatin is one of the most effective chemotherapeutic drugs for colon cancer treatment. However, the unavoidable toxicity, as well as the development of acquired resistance severely limited its clinical applicability. In combination with cisplatin, EGCG enhanced the effect of cisplatin-induced autophagy in DLD-1 and HT-29 colorectal cancer cells, as characterized by the accumulation of LC3-II protein, improving the increase in acidic vesicular organelles (AVOs), as well as the formation of autophagosome [60].

### 6.2. Oxaliplatin

Oxaliplatin, a third-generation platinum drug, which has received more recently its approval for metastatic CRC treatment [79], is broadly used alone or in combination with 5-FU and leucovorin in FOLFOX regimens.

Curcumin has been shown to increase the chemotherapeutic potential of oxaliplatin, to overcome chemoresistance while improving the side effect profile of the oxaliplatin regimens [80]. The combined regimen with curcumin and oxaliplatin had synergistic potential in cell lines with acquired resistance to oxaliplatin, leading to the reversion of their resistant phenotype [61]. Curcumin inhibited the oxaliplatin-induced activation of NF-κB and decreases the expression of NF-κB signaling cascade, including downregulation of CXCL8, CXCL1, and CXCL2. Furthermore, CXCL8 and CXCL1 gene silencing re-sensitized oxaliplatin-resistant cells through the inhibition of the Akt/NF-κB signaling pathway [61]. Another study reported that HCT-116 and HT-29 CRC cells treated with FOLFOX for 48 h resulted in 60–70% overall survival and the activation of both IGF-1R, as well as EGFR. The addition of curcumin for another 48 h resulted in increased growth inhibition and concomitant reduction of EGFR, HER-2, IGF-R1, and Akt, as well as downregulation of COX-2 and cyclin-D1 [81]. Furthermore, difluorinated curcumin, a novel curcumin analog, has been shown to re-sensitize drug-resistant mice modulating the miR-21-PTEN-Akt axis [63].

The role of stem cells (CSCs), a small sub-population of self-renewal epithelial cancer cells, in chemoresistance is well-documented [45]. The addition of curcumin to FOLFOX-surviving colorectal cancer cells resulted in a significant reduction in CSCs, as evidenced by the downregulation of CD44, CD166, and EGFR, as well as by CSCs ability to form anchorage-dependent colonies [82]. Moreover, curcumin-inhibited EMT via regulation of TGF-β/Smad2/3 signaling cascade indicated that curcumin could reverse oxaliplatin-resistance in CRC. The same study confirmed the result in vivo using HCT116/OXA xenograft mice, demonstrating that both the tumor volumes and weights and Smad2/3 levels were reduced when animals were treated with the combined regimens compared with those administered with oxaliplatin alone [62]. Consistent with these results, curcumin formulating with phosphatidylcholine (Meriva) showed the enhanced efficacy of oxaliplatin in a combined regimen both in vitro and in vivo [83].

The research conducted by Kaminski et al. evaluated the implication of resveratrol in improving the chemotherapeutic effect of oxaliplatin in the Caco-2 colon cancer cell line, as well as its likely role in the inflammatory response [64]. The combined treatment was able to reduce cell growth synergistically via caspase-3 activation, PARP breakage, and mitochondrial membrane potential depolarization. Furthermore, the combined treatment was effective in shaping an immune response, inhibiting the macrophages from becoming immunosuppressive. In another study, resveratrol sensitized CRC cells to oxaliplatin through up regulating the tumor suppressing microRNA, miR-34c, which silenced its target gene KITLG [65]. Furthermore, to determine the regimen’s in vivo effect, the same authors used a xenograft model in BALB/c athymic nude mice. The xenograft experiments showed that exposure to either resveratrol or oxaliplatin suppressed tumor growth, but the efficacy was evidently augmented when the two agents were used together. Moreover, a study conducted on HCT116 colon cancer cell line showed that resveratrol can reverse oxaliplatin-induced survivin suppression in HCT116 cells, restoring survivin expression at both mRNA and protein level, and pled for caution when using resveratrol with oxaliplatin in clinical practice [66]. Survivin, one of the smallest members of the inhibitor of apoptosis proteins (IAP) is related with poor prognosis and chemoresistance, has anti-apoptotic and pro-angiogenic properties, and it is known to be a target of oxaliplatin treatment in CRC [66,84].

Other promising natural compounds have been proposed in combination with oxaliplatin, such as betulinic acid, alanolactone, or piperlongumine, with promising results but yet only scarce research available. In oxaliplatin-resistant CRC cells treated with betulinic acid, Bcl-2 anti-apoptotic protein was down-regulated, whereas pro-apoptotic Bad was up-regulated [67]. Furthermore, alantolactone, a sesquiterpene lactone extracted from the roots of *Inula helenium* L., a Chinese herbal remedy, enhanced the effect of oxaliplatin on colorectal cancer HCT-116 cells through induction of ROS, activation of JNK and p38 MAPK apoptotic pathways [68]. Another novel therapeutic approach using oxaliplatin and piperlongumine, a natural product derived from the *Piper longum Linn* plant, was tested. Piperlongumine sensitized CRC cells to oxaliplatin by inducing ROS-dependent endoplasmic reticulum stress and mitochondrial dysfunction in CRC. Moreover, in vitro evidence was strongly supported by xenograft experiments in mice models [69].

## 7. Irinotecan

Irinotecan, a semisynthetic analog of camptothecin, is a DNA topoisomerase I inhibitor able of interfering with DNA replication and transcription. In its active, metabolized form (SN-38), irinotecan stabilizes the intermediate cleavage complexes formed between topoisomerase I and the single strand of DNA, preventing DNA ligation, and so enhancing cell death. In clinical practice, irinotecan represents a first-line therapeutic approach for advanced stages of colorectal cancer associated with invasion and metastasis [85]. Even though the addition of irinotecan to the standard regimens used for treating advanced metastatic CRC significantly improved the therapeutic outcomes [86], the response rate to these regimens is only in the range of 30–50% [87]. The primary reason for treatment failure is thought to be the tumor’s acquired drug resistance, a phenomenon that occurs in 90% of the patients with metastatic CRC [86]. Therefore, acquired resistance to irinotecan in patients with advanced CRC is still a major clinical issue. Only a limited number of studies have evaluated the interaction of bioactive dietary components with irinotecan and show promising results.

Curcumin and irinotecan are more effective at decreasing cell viability and increasing apoptosis and cell cycle arrest when administered simultaneously, in comparison to the single drug treatments. The observed effects of curcumin were via endoplasmic reticulum stress pathway, as well as through ROS generation [70]. In terms of re-sensitizing chemoresistant cells, promising data are available. Su et al. indicated that curcumin can efficiently attenuate chemoresistance of CRC cells by inducing apoptosis in an irinotecan-resistant cell line by significantly altering the expression levels of CSC identification markers. Moreover, curcumin up-regulated Bax pro-apoptotic protein expression, while down-regulating the anti-apoptotic Bcl-2 [71]. Likewise, Zhang et al. demonstrated that curcumin is an effective chemosensitizing agent able to reverse EMT in colorectal cancer. In irinotecan-resistant cells (LoVo/CPT-11R) treated with curcumin, E-cadherin expression was upregulated, whereas vimentin and N-cadherin expressions were downregulated [72]. In addition to the re-sensitizing potential, Ouyang et al. demonstrated that curcumin exerts a protective effect against irinotecan-induced intestinal mucosal injury mediated by the inhibition of NF-κB activation [73].

A recent study evaluated the combined approach with EGCG and irinotecan on migration, invasion, DNA damage, cell cycle, and autophagy of CRC cells. The authors demonstrated that the combination treatment had a stronger inhibitory effect than either agent alone and was able to prevent tumor cell migration and invasion. Furthermore, EGCG and irinotecan can synergistically inhibit the activity of topoisomerase I, leading to more extensive DNA damage [74].

## 8. Doxorubicin

Doxorubicin is a chemotherapy medication routinely used in the treatment of several cancers, including CRC regimens. It acts as a DNA topoisomerase II inhibitor, thus impairing DNA transcription and replication. However, it is associated with cardiac side effects, the most common drawback being the development of heart failure [88]. In the attempt of finding strategies for lowering the total dose administered, the combined treatment with resveratrol was proposed. The cotreatment of resveratrol and doxorubicin up-regulated pro-apoptotic gene Bax in colorectal HCT116 cell line enhancing apoptosis, while limiting the efflux of the drug via down-regulation of ABCB1 member of ABC proteins, and thus increases the intracellular concentration of the drug, as well as its therapeutic effect [75]. Likewise, in Caco-2-resistant colorectal cancer cells, the resveratrol-mediated inhibition of ABC-transporters efflux functions by altering mRNA levels of ABCB1/MDR1, MRP1, and BCRP [76]. In addition, the use of resveratrol at very low concentrations, in combination with doxorubicin, resulted in a dose reduction for doxorubicin while maintaining the same cytotoxic effect, thus overcoming the most important challenge associated with the administration of doxorubicin.

## 9. Conclusions and Further Perspectives

Plant-derived bioactive compounds represent strong candidates in order to overcome the increasing prevalence of acquired drug-resistance in cancer therapy. When administered in combination with conventional chemotherapeutic agents, they often yield augmented antitumor effects and are able to block and/or reverse the acquired drug-resistance mechanisms. Considering the research papers presented in this review (summarized in Table 1), bioactive dietary components can enhance the chemotherapeutic effect of the drugs and reduce chemoresistance by decreasing efflux proteins belonging to ABC family transporters, promoting apoptotic cell death, as well as reversing EMT. One of the major problems in the clinical translation is related to the limited bioavailability of most of the compounds, such as curcumin or resveratrol, since both are fast metabolized and eliminated by the body, making the maintenance of the therapeutically appropriate concentration in the circulation challenging. The advance of nanotechnology opens numerous possibilities for improving the therapeutic effects of these plant-derived bioactive compounds. By lowering the particle size, altering the surface, and entrapping the compound with various nanocarriers, a well-formulated nano approach increases bioavailability and bioactivity as reported in extensive detail in recent reviews in the case of curcumin [89,90], resveratrol [91] or EGCG [92]. As further knowledge is gained in the formulation of novel drug delivery systems, is essential that these natural compounds undergo clinical evaluation through a large number of clinical trials to validate the effectiveness observed in vitro, as well as in animal models. In addition, it is necessary to establish the safety profile of each compound when administered in cancer patients. Despite the hurdles that lie ahead, the potential of these plant-derived bioactive components is enormous, and it is well worth the collaborative effort of the scientific community for the benefit of oncological patients.

## Figures and Tables

**Table 1:** Main in vitro and in vivo effects of chemotherapeutic agents in combination with plant-derived bioactive components against CRC.

Drug	Plant-Derived Bioactive Component	Experimental Model	Main Effect	Molecular Target	Ref.
**5-FU**	Curcumin	HCT-8 HCT-8/5-FU (5-FU-resistant)	Reversal effects on the multidrug resistance	↓ P-gp HSP-27	[52]
		SW-480	Enhance the therapeutic effects of 5-FU Induction of ROS	↓ Sp1, Sp3, Sp4, miR-27a	[53]
		HCT116/ HCT116-5FUR SW480/SW480-5FUR	Suppressed EMT	↑ miR-200b, miR-200c, miR-141, miR-429, miR-101 ↓ BMI1, SUZ12, EZH2 mRNA	[54]
	EGCG	DLD-1	Synergistic growth suppression, apoptosis	-	[24]
		HCT-116	Promotes cancer cell apoptosis and suppressed EMT	↓ GRP78, MDR1, Bcl-2 ↑ miR-155-5p, caspase-3 PARP, Bad	[23]
	Resveratrol	DLD-1	Synergistic growth suppression, apoptosis	miR-34a/E2F3/Sirt1 cascade	[24]
		DLD1 HCT116	Enhanced the antiproliferative potential of 5-FU Augmented 5-FU pro-apoptotic effect Repressed EMT transition	↓ Akt and STAT3 pathways ↓ slug and vimentin	[57]
		HT-29 SW-620 CRC	Enhanced ROS and lipid peroxides	↓ AKT, STAT3	[58]
		HCT116/HCT116R in 3D-alginate tumor microenvironment	Suppressed EMT	↓ TNF- β, vimentin, slug ↑ E-cadherin	[59]
**Cisplatin**	EGCG	DLD-1 HT-29	Synergistic effect on inhibition of cell proliferation and induction of cell death	↑ LC3-II	[60]
**Oxaliplatin**	Curcumin	HT29/HTOXAR3 DLD1/DLDOXAR3 LoVo/LoVOXAR3	Re-sensitized oxaliplatin-resistant cells	↓ NF-κB, CXCL8, CXCL1, CXCL2 ↓ Akt pathway	[61]
		HCT116/OXA xenograft mice	Reverse oxaliplatin resistance-reduced tumor weight and volume	↓ Smad2/3	[62]
	Difluorinated-Curcumin	HCT116 xenograft mice	Re-sensitize drug-resistant mice	miR-21-PTEN-Akt axis	[63]
	Resveratrol	Caco-2	Reduce cell growth immunomodulator	↑ caspase-3, PARP	[64]
		HT-29 HCT-116	Sensitized cells to oxaliplatin	↑ miR-34c ↓ KITLG	[65]
		HCT116 xenograft mice	Augmented efficacy on suppressing tumor growth	-	[65]
		HCT116	Antichemosensitizing effect	↑ survivin	[66]
	Betulinic acid	SNU-C5/OXT-R	Sensitized cells to oxaliplatin	↓ Bcl-2 ↑ Bad	[67]
	Alanolactone	HCT-116	Induction of ROS Enhanced the effect of oxaliplatin	JNK, p38 MAPK apoptotic pathways	[68]
	Piperlongumine	HCT-116 LoVo	Sensitizes cells to oxaliplatin Enhances oxaliplatin-associated ROS production	↓ Bcl-2 ↑ Bax, ER-stress- associated proteins (eIF2α, ATF4, CHOP)	[69]
**Irinotecan**	Curcumin	LoVo HT-29	Enhanced the effects of irinotecan in inhibiting colorectal cancer cell viability Enhanced the anti-tumor activity of irinotecan through reactive oxygen species generation	-	[70]
		LoVo/CPT-11 (irinotecan-resistant cells)	Significantly attenuated chemoresistance to irinotecan through induction of apoptosis of CSCs	↓ CD44, EpCAM, CD24, Bcl-2 ↑ Bax	[71]
		LoVo/CPT-11R	Suppressed EMT	↑ E-cadherin ↓ vimentin, N-cadherin	[72]
		Irinotecan-treated BALB/c nude mice	Protective effect against irinotecan-induced intestinal mucosal injury	↓ NF-κB	[73]
	EGCG	RKO HCT116	Stronger inhibitory effect on tumor cells Prevention of migration and invasion S or G2 phase arrest induction of apoptosis	↓ topoisomerase I	[74]
**Doxorubicin**	Resveratrol	HCT116	Sensitize colorectal cancer cells to doxorubicin via facilitating apoptosis and enhancing intracellular entrapment	↑ Bax ↓ P-gp	[75]
		Multidrug-resistant Caco-2	Inhibition of ABC-transporters’ efflux functions	↓ P-gp, MRP1, BCRP, CYP3A4, GST, hPXR mRNA	[76]

↓—increased, ↑—decreased.

## Data Availability

Not applicable.
